# Conventional DNA-Damaging Cancer Therapies and Emerging cGAS-STING Activation: A Review and Perspectives Regarding Immunotherapeutic Potential

**DOI:** 10.3390/cancers15164127

**Published:** 2023-08-16

**Authors:** Jordan D. Lewicky, Alexandrine L. Martel, Mukul Raj Gupta, René Roy, Galaxia M. Rodriguez, Barbara C. Vanderhyden, Hoang-Thanh Le

**Affiliations:** 1 Health Sciences North Research Institute, 56 Walford Road, Sudbury, ON P3E 2H2, Canada; jlewicky@hsnri.ca (J.D.L.); amartel@hsnri.ca (A.L.M.); 2 Glycosciences and Nanomaterial Laboratory, Université du Québec à Montréal, Succ. Centre-Ville, Montréal, QC H3C 3P8, Canada; guptamukulraj@gmail.com (M.R.G.); roy.rene@uqam.ca (R.R.); 3 Cancer Therapeutics Program, Ottawa Hospital Research Institute, 501 Smyth Rd., Ottawa, ON K1H 8L6, Canada; garodriguez@ohri.ca (G.M.R.); bvanderhyden@ohri.ca (B.C.V.); 4 Department of Cellular and Molecular Medicine, University of Ottawa, 451 Smyth Rd., Ottawa, ON K1H 8M5, Canada; 5 Medicinal Sciences Division, NOSM University, 935 Ramsey Lake Road, Sudbury, ON P3E 2C6, Canada; 6 School of Natural Sciences, Laurentian University, 935 Ramsey Lake Road, Sudbury, ON P3E 2C6, Canada

**Keywords:** DNA damage response, cGAS-STING, cancer immunotherapy, chemotherapy, radiation, tumor microenvironment, cGAMP, chemoimmunotherapy, cytosolic DNA

## Abstract

**Simple Summary:**

The cGAS-STING cellular signaling pathway is a key member of the DNA damage response, whose role is to repair the DNA damage that occurs naturally during the life of a cell. Interestingly, cGAS-STING is known to promote immune responses against tumors, and is being explored for its potential use in cancer immunotherapy applications. The DNA damage caused by traditional cancer treatments such as radiation and chemotherapy is one of the main ways in which cancer cells are eradicated, and is increasingly being linked with cGAS-STING activation. In this review, we summarize the many reports of cGAS-STING activation by different conventional cancer therapies, highlighting the roles of their targets in the DNA damage response. As part of the review, we discuss an emerging “chemoimmunotherapy” concept where the DNA-damaging activity of these conventional therapies can potentially be exploited for its beneficial stimulation of anticancer immune responses by way of cGAS-STING activation. The potential advantages of such an approach are highlighted, and it becomes clear that targeted nanoparticle delivery systems will be critical in minimizing the associated immunotoxic and inflammatory activities of the entrapped chemotherapeutics.

**Abstract:**

Many traditional cancer treatments such as radiation and chemotherapy are known to induce cellular DNA damage as part of their cytotoxic activity. The cGAS-STING signaling axis, a key member of the DNA damage response that acts as a sensor of foreign or aberrant cytosolic DNA, is helping to rationalize the DNA-damaging activity of these treatments and their emerging immunostimulatory capacity. Moreover, cGAS-STING, which is attracting considerable attention for its ability to promote antitumor immune responses, may fundamentally be able to address many of the barriers limiting the success of cancer immunotherapy strategies, including the immunosuppressive tumor microenvironment. Herein, we review the traditional cancer therapies that have been linked with cGAS-STING activation, highlighting their targets with respect to their role and function in the DNA damage response. As part of the review, an emerging “chemoimmunotherapy” concept whereby DNA-damaging agents are used for the indirect activation of STING is discussed as an alternative to the direct molecular agonism strategies that are in development, but have yet to achieve clinical approval. The potential of this approach to address some of the inherent and emerging limitations of cGAS-STING signaling in cancer immunotherapy is also discussed. Ultimately, it is becoming clear that in order to successfully employ the immunotherapeutic potential of the cGAS-STING axis, a balance between its contrasting antitumor and protumor/inflammatory activities will need to be achieved.

## 1. Introduction

Traditional strategies in the treatment of cancer include surgical resection, radiation, chemotherapy, or some combination thereof. Surgery is not an option for many cancers, and in those cases where it is possible, success relies on both the ability of the patient to survive the surgery and the ability of the surgeon to completely remove all of the diseased tissues [1]. Radiation and chemotherapy serve to eradicate malignant cells; however, these treatments are not selective, causing off-target toxicity in healthy tissues that leads to significant undesired side effects and the potential for highly dangerous immunosuppression [2]. In addition, in cases where chemotherapy fails to completely eradicate the cancer, there is significant risk for the development of resistance to the treatment, increasing the risk of both metastasis and disease relapse [3].

Cancer immunotherapy, which has attracted considerable attention in recent years, aims to sensitize the patient’s immune system to the disease for its selective eradication [4]. Various immunotherapy strategies continue to be explored, with several having gained clinical approval [5]. Most prevalent among the clinically approved immunotherapies are checkpoint inhibitors (ICIs), which modulate the aberrant regulation that prevents the immune system from attacking cancer cells [6]. Unfortunately, the benefits of ICIs are limited to specific cohorts of patients who have inherently higher T cell levels at the tumor site [7]. ICIs also suffer from other limitations, including the development of treatment resistance, and toxicity that arises from the disruption of the natural homeostatic balance of the immune system [8]. Ultimately hindering the success of any cancer immunotherapy is the tumor microenvironment (TME), which dynamically regulates therapeutic responses and contributes to treatment resistance [9]. Large populations of immunosuppressive cells, including tumor-associated macrophages (TAMs) and regulatory T cells (Tregs), contribute to a non-inflamed “cold” TME, which limits the infiltration and activity of T cells within the tumor [10]. In addition, down-regulation of major histocompatibility complex class 1 (MHC-1) immune ligand expression on the surface of cancer cells significantly hampers the potential for immunosurveillance [11,12]. Thus, there is a considerable need for novel immunotherapy strategies that can induce an inflamed “hot” TME that promotes cytotoxic T lymphocyte (CTL) and natural killer (NK) cell responses by way of balancing TME immunosuppressive effects, increasing tumor immunogenicity via enhanced tumor-associated antigen presentation, and increasing the trafficking and infiltration of T cells into the tumor [13,14].

**Figure 1:**
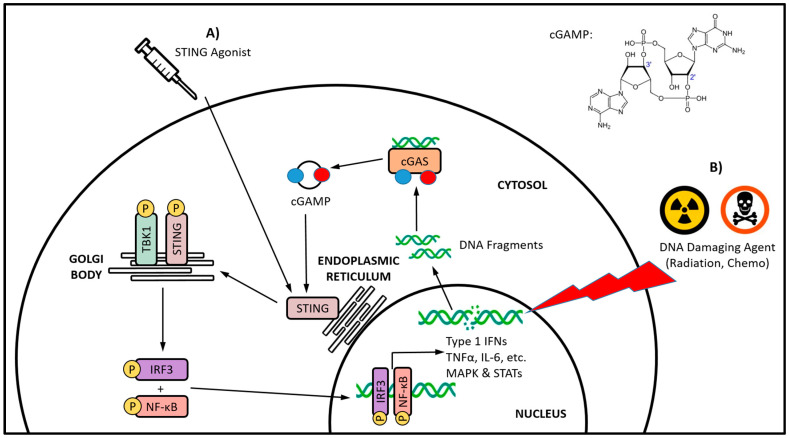
The cGAS-STING axis signaling and a comparison of direct versus indirect STING activation. Direct STING activation (**A**) is being targeted with STING agonists, whereas indirect activation (**B**) occurs via cGAS sensing of cytosolic DNA fragments, and its subsequent endogenous synthesis of the canonical CDN STING agonist cGAMP. The DNA damage induced by traditional cancer therapies, such as radiation and certain chemotherapeutics, has been increasingly linked with indirect STING activation. Whether directly or indirectly activated, the translocation of STING from the endoplasmic reticulum to the Golgi body initiates a series of phosphorylation-based signaling events culminating in the activation of the IRF3 and NF-κB transcription factors, and subsequent production of type I IFNs and various other proinflammatory cytokines.

The stimulator of interferon genes (STING) cellular signaling pathway is emerging as a promising immunotherapeutic target to combat the immunosuppressive TME [15]. The STING pathway is an important player in the DNA damage response (DDR) and is involved in monitoring the cytosol of cells for the presence of foreign or damaged DNA, a distress signal that arises from a multitude of potential threats [16]. The STING protein is activated by the cyclic dinucleotide (CDN) 2′3′-cyclic guanosine monophosphate–adenosine monophosphate (cGAMP), a second messenger molecule that is produced by cGAMP synthase (cGAS) after it detects the presence of cytosolic double-stranded DNA (dsDNA) [17,18]. The recognition of dsDNA by cGAS is mostly sequence-independent, with the minimal length for cGAS activation being 20–40 base pairs and varying between species [19,20]. Upon binding cGAMP, the STING homodimer undergoes extensive conformational rearrangements and oligomerization, and ultimately translocates from the endoplasmic reticulum to the Golgi body (Figure 1) [21,22,23,24]. The recruitment and activation of TANK-binding kinase 1 (TBK1) by autophosphorylation leads to the phosphorylation of STING [25,26]. The resulting STING–TBK1 complex phosphorylates interferon regulatory factor 3 (IRF3), leading to its homodimerization and nuclear translocation, where it induces target gene expression [27,28]. The downstream effects of STING pathway activation are variable, and are dependent on the particular cell type, as well as the intensity and duration of activation [29]. However, a characteristic of STING pathway signaling is the secretion of type I IFNs [30,31,32]. In some settings, STING signaling can also be associated with the activation of nuclear factor κB (NFκB), mitogen-activated protein (MAP) kinases, and signal transducer and activator of transcription (STAT) transcription factors [33,34,35,36,37].

**Figure 2:**
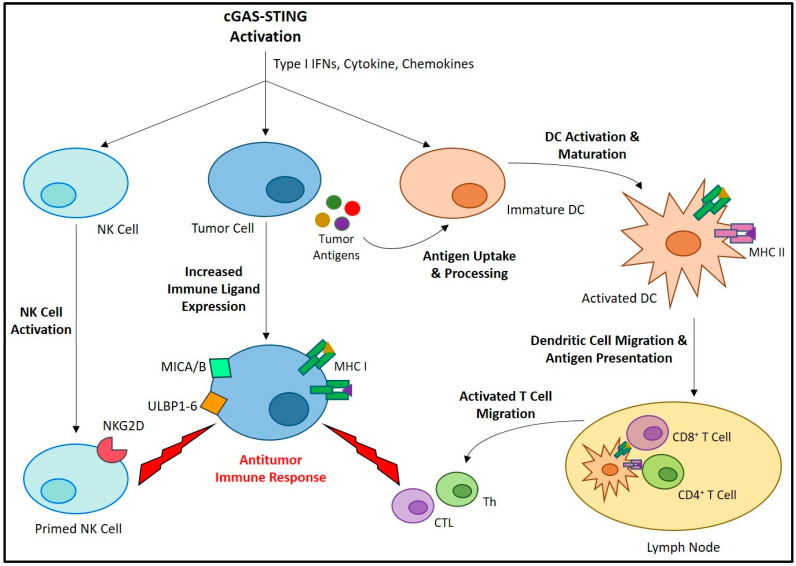
The role of the cGAS-STING axis in promoting anticancer immunity. The activation of cGAS-STING and the resulting type I IFNs, cytokines, and chemokines induce a variety of cell-specific responses that all contribute to antitumor immune activity. Activation of immature DCs leads to increased antigen uptake/processing, maturation, and lymph node migration. Here, the DCs present antigens to naïve T cells, and potentiate their differentiation into Th and CTL, which migrate and infiltrate the tumor site where they carry out antitumor responses. A similar process occurs in NK cells, in which their priming leads to an increased expression of immune receptors, including NKG2D. These cellular immune responses are promoted by requisite increases in tumor cell immune ligand expression, including MHC-1 for CTL recognition, as well as MHC class 1 chain-related protein A and B (MICA/B) and UL16-binding proteins 1-6 (ULBP1-6) for recognition by NKG2D.

Type I IFNs directly regulate the transcription of over 100 genes that influence many key aspects of cell survival and immunity [38]. In the cancer setting, type I IFNs have been shown to directly inhibit the proliferation of tumor cells [39,40,41], and disrupt tumor vasculature [42,43]. While the production of type I IFNs is a critical component of STING signaling for promoting antitumor immunity, the other IFN-independent signaling pathways downstream of STING activation also play a key role in cancer immune regulation [44]. Taken together, the cancer immunotherapeutic potential of STING signaling arises from its capacity to promote a wide array of antitumor immune responses (Figure 2) [45]. STING activation promotes the maturation of professional antigen-presenting cells (APCs), leading them to express various costimulatory molecules and pro-inflammatory cytokines required for T cell priming and activation [46,47,48,49]. Both T helper lymphocyte (Th) [50,51] and CTL [52,53] responses are known to be enhanced by STING-induced APC maturation [54,55]. Furthermore, a balanced type 1/type 2 (Th1/Th2), or even Th1 biased phenotype [56] have been reported in response to STING-induced IFN signaling, thereby promoting M1 macrophage polarization [57,58]. Compared to M2 macrophages, which are known to be immunosuppressive, M1 macrophages are more supportive of antitumor immune responses [59,60,61]. STING-induced pro-inflammatory cytokines, including interleukin 6 (IL-6) and tumor necrosis factor alpha (TNF-α), as well as reactive nitrogen and oxygen species (ROS), can promote M1 macrophage polarization and the repolarization of immunosuppressive TAMs [59,62,63]. STING signaling also generates a chemokine gradient, including CXCL10, CCL5, and CXCL9, that can guide the recruitment and activation of T cells [52,64,65] and NK cells [66] within the tumor. STING signaling promotes MHC-1 expression on the cancer cell surface that is required for CTL recognition and eradication [67]. Furthermore, STING signaling has been shown to upregulate expression of the ligands of NK group 2, member D (NKG2D), an NK cell-specific immunoreceptor necessary for the recognition and elimination of cancer cells [68].

The promising immunostimulatory potential of the STING pathway has fueled considerable efforts geared towards the development of STING agonist therapeutics (Figure 1A) (reviewed in [69]). STING agonists have shown success for generating antitumor immunity against a wide range of cancer types in preclinical research, prompting numerous clinical trials (reviewed in [15]). However, both the safety and efficacy of STING agonists are limited by numerous pharmacological and drug delivery challenges, including metabolic stability issues, low cellular uptake/intracellular delivery, and the potential for immune-related adverse events including cytotoxicity [70,71]. Another major concern in the development of STING agonists is systemic administration, and the lack of tissue or cell specificity. In addition to cancer cells, STING is expressed in many other cell types that may have adverse responses impacting therapeutic outcomes [72]. While STING activation in extratumoral cell populations may result in antitumor effects, the ideal location for STING activation is within the tumor site, where it can generate the necessary local immunostimulatory effects [73]. Restricting systemic-wide STING activation is necessary to minimize nonspecific systemic inflammatory responses [74]. Ultimately, while STING pathway agonists offer considerable promise for cancer immunotherapy, none have yet gained clinical approval.

Despite the promising immunotherapeutic potential of the STING pathway for cancer treatment, there are several critical and inherent concerns. The duration of STING pathway stimulation is an important consideration, as it can drastically influence the balance of immunological outcomes. Localized and acute activation of the STING pathway supports an appropriate level of immune activation for cancer eradication. However, chronic STING signaling is implicated in a variety of inflammation-driven diseases [75]. In addition, prolonged STING pathway activation can also lead to cancer development and metastasis [76,77,78,79]. While the production of type I IFNs is a critical component of STING-signaling-induced antitumor immunity, recent studies have suggested that type I IFNs may actually impair anticancer immunity and cause treatment resistance. For example, IFN-β has been shown to increase the levels of programmed cell death ligands 1 (PD-L1) and 2 (PD-L2), which are known to contribute to immune escape by cancer cells [80,81]. Type I IFNs have also been shown to contribute to unexpected immune toxicity during cancer immunotherapy [82]. In addition, the other IFN-independent signaling pathways downstream of STING activation also play a key role in immune regulation, and can contribute to tumor immune evasion [44,83].

It has been known for some time that traditional cancer treatments such as radiation and chemotherapy have beneficial immunostimulatory effects that continue to be reported [84]. These cancer treatments stimulate aspects of both the innate and adaptive immune systems via several different mechanisms. The cytotoxic activity of radiation and chemotherapy have been linked to the induction of immunogenic cell death, which leads to the release of specific signals that trigger the phagocytosis of cellular debris and the maturation of APC [85]. In addition, these traditional cancer treatments have been reported to alleviate tumor-induced immunosuppressive mechanisms [86,87,88], and cause transient lymphodepletion that allows for localized immune cell replenishment [89]. Mechanistically, common to the majority of these traditional cancer treatments is their DNA-damaging activity, which ultimately leads to their cytotoxicity. Opposing this activity is the DDR, which is a highly organized network of interconnected components that are responsible for the repair of damaged DNA and the maintenance of genomic stability [90]. Defects in the DDR lead to an imbalance between DNA damage and repair that can drive tumorigenesis, inflammatory cytokine secretion, and aberrant immune responses [91]. Emerging evidence is outlining the critical link between the DDR and antitumor immunity, in that it shapes the innate immune response and how the adaptive immune system is recruited to tumors [92]. Most immune-related DDR components and immune responses converge upon the STING-IFN signaling pathway [93]. It is not surprising then, that many of the traditional, DNA-damaging cancer treatments are being linked with indirect and iatrogenic STING activation (Figure 1B). However, the significance of said STING activation in cancer patients and potential immunotherapeutic applications are currently unclear.

In this review, we summarize the many different conventional cancer therapies reported to activate the cGAS-STING signaling axis. We discuss the targets of these therapies in relation to their role and function in the DDR. In our summary, we pay particular attention to the doses used, given the potential of these therapies to be toxic and damaging to the immune system. Despite this potential of adverse immunological effects, an emerging concept of a “chemoimmunotherapy” strategy in which DNA-damaging agents are used for an indirect activation of STING is discussed, which aligns with our own results that are included in the review. The potential of this approach to address some of the inherent and emerging limitations of the cGAS-STING axis in cancer immunotherapy is considered, where it becomes evident that low doses of the DNA-damaging agent will be required to avoid detrimental immunotoxicity, and will need to be supported with the use of targeted nanoparticle delivery systems.

## 2. Conventional Cancer Therapies and cGAS-STING Activation

In general, the different classes of conventional cancer therapies that have been linked with activation of the cGAS-STING signaling axis can be classified into three categories (Table 1), the chemical structures of which are shown in the Supplemental Table S1: (i) microtubule-targeting antimitotic agents, (ii) inhibitors of DDR enzymes that damage DNA indirectly, and (iii) agents that directly damage DNA.

### 2.1. Antimitotic Agents

Microtubule-targeting agents (MTAs), such as Taxol, have been used extensively for the treatment of rapidly dividing cancers, and cause cell death by inducing mitotic arrest and eventual apoptosis [121]. While MTAs do not directly interact with DNA or members of the DDR, their activity has been linked with the induction of limited degrees of DNA damage resulting from the partial activation of apoptosis and its associated nucleases [122,123]. MTAs are also known to disrupt the intracellular trafficking of various DNA repair proteins, thus augmenting the toxicity of DNA-damaging chemotherapies which is the basis for their combination as a common anticancer regimen [124].

Zierhut et al. demonstrated that Taxol-induced mitotic arrest in the HeLa cervical cancer cell line resulted in a slow accumulation of IRF3 phosphorylation in a cGAS-dependent manner [94]. This triggered apoptosis via the transcription-independent release of BCL-xL-dependent suppression of mitochondrial outer membrane permeabilization. The authors went on to demonstrate that histone-wrapped nucleosomic DNA had a higher affinity for cGAS than naked DNA, and prevented cytoplasmic chromosomal DNA from activating cGAS during normal mitosis by competitive inhibition. In addition, the cGAS expression level was shown to correlate with Taxol sensitivity in a panel of breast cancer cell lines, and promoted the response to Taxol in a mouse xenograft model of cervical cancer. Lohard et al. showed that Taxol induced DNA micronuclei in breast cancer cell lines and patient-derived mouse xenografts that led to the downstream activation of the cGAS-STING pathway [95]. The resulting secretion of type I IFN and TNF-α formed a proapoptotic paracrine secretome, which triggered NOXA expression in neighboring cells, and increased their sensitivity to the inhibition of BCL-xL. Hu et al. demonstrated that cytotoxic doses of Taxol induced cGAS-positive micronucleation and subsequent STING signaling in various triple-negative breast cancer (TNBC) cell lines [96]. The resulting cytokines and chemokines from this response were able to induce a M1-like polarization of THP-1 derived macrophages in vitro. The authors further showed that similar doses of other MTAs, including vinorelbine and eribulin, also induced cGAS-positive micronucleation; however, evidence of subsequent downstream STING signaling was not provided. In a somewhat contradictory report to that of Hu et al., Fermaintt et al. showed that cytotoxic doses of eribulin, but not Taxol, induced cGAS-STING-depended expression of type I IFNs in both myeloid and TNBC cells in vitro [97]. The cGAS-STING pathway activation by eribulin was further demonstrated to be mediated by the accumulation of cytoplasmic mitochondrial DNA. Overall, while these results suggest there may be the potential that MTAs, in general, may be able to activate the cGAS-STING signaling pathway via the DNA damage they induce, further work will need to confirm this.

### 2.2. DDR Inhibitors

#### 2.2.1. Topoisomerase Inhibitors

Topoisomerases are enzymes that catalyze changes in the intertwined double-helical structure of DNA [125]. They are critical for transcription, replication, and DNA repair [125,126]. There are two classes of topoisomerases, which differ in the mechanism by which they facilitate the transient breakage of DNA strands. Topoisomerase I (Topo I) catalyzes changes in DNA structure via single-strand breaks (SSB), whereas topoisomerase II (Topo II) functions through double-strand breaks (DSB) [127]. Topoisomerase inhibitors, such as camptothecin, topotecan, doxorubicin, and daunorubicin, are used as both antibacterials and chemotherapeutics, where they interfere with the catalytic cycle of the enzyme and lead to elevated levels of covalent enzyme–DNA complexes [128]. Ultimately, irreversible DNA damage results in having highly prevalent DSB occurring upon collision of the replicative machinery with drug-stabilized topoisomerase–DNA complexes [129]. Lymphodepletion is one of the most common side effects associated with topoisomerase inhibitors, and may result in detrimental immunosuppression [130].

Pépin et al. showed that low doses of the topo I inhibitor camptothecin induced minor levels of DNA damage that resulted in a strong cGAS-STING-dependent antiviral gene response in viral oncogene-expressing mouse embryonic fibroblast cells [98]. The viral oncogenes were further shown to potentiate the leakage of damaged DNA into the cytoplasm, which was critical for subsequent cGAS recruitment. As part of a chemoimmunotherapy strategy, Cao et al. developed a hybrid prodrug made up of both camptothecin and the alkylating agent cisplatin to specifically promote DNA damage-associated STING activation in tumor cells [99]. Encompassing the chemotherapy hybrid was a ROS-sensitive polymeric nanoparticle, which improved the delivery, DNA-damaging activity, and subsequent cGAS-STING pathway activation in several cancer cell lines in vitro. In addition, the system activated T lymphocytes, promoted CD8+ T cell transformation into memory cells, and induced strong adaptive antitumor immune responses in an in vivo murine colorectal cancer model. The authors did not demonstrate the degree to which degree each DNA-damaging agent contributed to the overall cGAS-STING-driven response. Working with a camptothecin derivative (7-ethyl-10-hydroxycamptothecis, SN38), Zhao et al. demonstrated that the compound induced DNA damage in tumor cells which resulted in the passage of DNA-containing exosomes to APCs and subsequent cGAS-STING pathway activation [100]. The SN38 was incorporated into a polymeric conjugate capable of self-assembly into nanoparticles, which not only reduced the toxicity of the drug, but also improved its ability to activate the cGAS-STING pathway and induce strong antitumor immune responses in an in vivo murine model of breast cancer. Another example of a camptothecin derivative is topotecan, which was shown by Kitai et al. to trigger STING-dependent DC activation and cytokine release [101]. Various cancer cell lines treated with cytotoxic doses of the drug released DNA-containing exosomes that were taken up by DCs in a paracrine fashion. In an in vivo mouse model of breast cancer, topotecan induced significant antitumor immune responses that were driven by the infiltration of activated DCs and CD8+ T cells.

Luthra et al. demonstrated that non-cytotoxic doses of the topo II inhibitors doxorubicin and daunorubicin triggered a cGAS-STING-dependent IFN induction in HEK 293T and A549 cells expressing the Ebola virus VP35 protein [102]. The response induced by these compounds was further shown to inhibit Ebola virus replication in vitro. With cytotoxic doses of the topo II inhibitor etoposide, Wang et al. demonstrated an induction of DNA damage that resulted in both STING-dependent type I IFN signaling, and NF-κB activation in various cancer cell lines in vitro [103]. They went on to demonstrate activated antitumor T cell responses, both in vitro and in vivo, which potentiated the antitumor efficacy of anti-PD1 antibody treatments in several different mouse tumor models. Interestingly, Dunphy et al. demonstrated that non-cytotoxic dose of etoposide induced an early non-canonical activation of STING in HaCaT keratinocytes that was independent of cGAS [104]. No production of the cGAMP second messenger, nor STING phosphorylation or translocation from the ER were observed. The DNA repair proteins ataxia telangiectasia mutated (ATM) and poly(ADP-ribose) polymerase 1 (PARP1) were required for the induction of this innate immune response, together with the DNA binding protein gamma-interferon-inducible protein 16 (IFI16), p54, and the E3 ubiquitin ligase TRAF6. The resulting signaling complex yielded an alternative STING-dependent expression program predominantly skewed towards NFκB signaling, with only a minor contribution from IRF3.

#### 2.2.2. PARP Inhibitors

The PARP family of proteins are involved in a variety of cellular processes that include chromatin remodeling, as well as the transcription, replication, and repair of DNA damage [131]. The main role of PARP in the DDR is the recruitment of other DNA-repairing enzymes via the synthesis of a polymeric ADP-ribose chain signal after detecting DNA damage [132]. PARPs are involved in the base-excision repair of DNA SSB and the resection of DSB via both HR and non-homologous end joining mechanisms [133,134]. The DNA repair activity of certain PARP proteins is exploited for survival by certain cancers that are defective in homologous repair (HR) mechanisms, and are therefore sensitive to its inhibition [135]. Several PARP inhibitors (PARPi), such as olaparib, rucaparib, and talazoparib, have been approved as targeted chemotherapeutics for the treatment of cancers with mutations in the essential HR genes breast-cancer-associated 1 and 2 (BRCA1 and BRCA2), where the resulting accumulation of DNA DSB leads to genomic instability and eventual cell death [136]. While PARP-inhibitor-induced DNA damage is known to have beneficial immunostimulatory effects, which include the activation of the cGAS-STING pathway as discussed below, PARP inhibitors also upregulate immunosuppressive PD-L1 [132].

Chabanon et al. demonstrated that cytotoxic doses of the PARPis olaparib and rucaparib induced DNA damage and cGAS-STING-dependent type I IFN signaling in both a BRCA1-deficient TNBC cell line, and lung cancer cells lacking the excision repair cross-complementation groups 1 (ERCC1) protein, a common DDR defect in non-small-cell lung cancers (NSCLC) [105]. They also showed that the PARPis modulated the IFN-γ-induced PD-L1 expression in both NSCLC cell lines and patient tumor cells, an effect that was enhanced by ERCC1 deficiency. With cytotoxic doses of olaparib, Ding et al. showed that PARP inhibition induced strong local and systemic antitumor immune responses involving both CD4+ and CD8+ T cells in mice bearing BRCA1-deficient ovarian tumors [106]. The authors demonstrated that the effect was driven by a STING-dependent type I IFN response from APCs that sensed either DNA fragments or cGAMP induced in the tumor cells. Using a genetically engineered mouse model of TNBC, Pantelidou et al. showed that Olaparib induced CD8+ T cell infiltration and activation that resulted in strong antitumor immunity [107]. This response was diminished when CD8+ T cells were depleted, and was shown to be dependent on paracrine DC activation from cGAS-STING signaling in tumor cells. Using olaparib at doses in the IC50 range, the authors demonstrated that this effect was more prominent in HR-deficient than HR-proficient TNBC cells in vitro, and further confirmed this observation in vivo. Reisländer et al. demonstrated a dose-dependent induction of DNA DSB with olaparib in a H1229 NSCLC model possessing an inducible depletion of BRCA2 [108]. The BRCA2-deficient cells were more susceptible to olaparib induced DNA damage, which resulted in a rapid activation of an innate immune response involving increases in the mRNA levels of several interferon-stimulated genes (ISG). It is suggested that this response is conceivably from cGAS-STING activation; however, no direct evidence was provided. Shen et al. showed that cytostatic doses of talazoparib generated cytosolic dsDNA and activated cGAS-STING signaling in various gynecological cancer cell lines in vitro [109]. This activity was further confirmed in vivo in a mouse ID8 ovarian cancer model where strong antitumor immunity was induced independent of BRCA status. The authors further showed that talazoparib treatment triggered ISG expression in orthotopic xenograft mouse models of BRCA1-deficient TNBC and BRCA2-deficient colorectal cancer.

#### 2.2.3. Ataxia Telangiectasia and Rad3-Related (ATR) and ATM Inhibitors

ATR and ATM are critical signaling kinases in the DDR that are activated in response to DNA SSB and DSB, respectively [137,138]. They have pivotal roles in coordinating the DDR and the cell cycle to prevent replications stress. ATR is recruited to replication protein A (RPA)-coated single-stranded DNA via the ATR-interacting protein (ATRIP) [139,140], where it activates its major downstream effector, checkpoint kinase 1 (CHK1), which triggers intra-S and G2/M phase checkpoints [141]. ATM is recruited to DSB by the MRN complex [142], where it activates p53 by both phosphorylating it (Ser 15) and eliminating the inhibitory binding of the E3 ubiquitin ligase MDM2, which inhibits CDK2/cyclin E to induce cell cycle arrest at the G1 phase checkpoint [137]. Many chemotherapeutic agents induce an activation of ATR, which has been the rationale for its therapeutic inhibition [143,144].

While ATR and ATM inhibitors do not damage DNA directly, they have been shown to modulate immune responses induced by both radiotherapy [110,111,145,146] and DNA-damaging chemotherapies [147,148]. The combination treatment of radiotherapy and ATR or ATM inhibition was shown to induce both type I and type II IFN-associated gene responses, as well as increase CD8+ T cell infiltration [110,111,145,146]. There is emerging evidence that this activity may be due, in part, to cGAS-STING activation. Sheng et al. showed that the ATR inhibitor ceralasertib (AZD6738) increased the degree of radiotherapy-induced CD8+ T cell infiltration and activation in a mouse xenograft model of hepatocellular carcinoma, while also decreasing the immunosuppressive effects of the radiation on numbers of intratumoral Tregs and exhausted T cells [110]. The authors also showed that the addition of ATR inhibitor to an anti-PDL1 radioimmunotherapy program produced a cGAS-STING-dependent synergistic effect with increased infiltration, proliferation, and IFN-γ production from tumor infiltrating CD8+ T cells, as well as decreased numbers of tumor resident Tregs and exhausted T cells. Using the ATM inhibitor KU60019, Zhang et al. demonstrated a cooperative activity with radiotherapy to induce type I IFN signaling in human and mouse pancreatic cancer cell lines in a manner that was independent of cGAS-STING, but dependent on both TBK1 and the proto-oncogene tyrosine protein kinase SRC [111]. The combination of ATM inhibition and radiotherapy was further shown to increase PDL-1 expression and tumor sensitivity to anti-PDL-1 therapy in a mouse model of pancreatic cancer, accompanied by an increased tumoral infiltration of CD8+ T cells that established immunological memory. Hu et al. showed a potent cGAS-STING pathway activation with the ATM inhibitors AZD1390 and KU55933 in both human breast cancer and mouse melanoma cells [112]. The authors linked the resulting increase in phosphor-TBK1 levels and ISG expression with down-regulation of mitochondrial transcription factor A, and subsequent cytoplasmic leakage of mitochondrial DNA.

#### 2.2.4. RNA Polymerase Inhibitors

RNA polymerases (RNAPs) are key to the transcription process, the continuity of which can be disrupted by lesions in the DNA template that result from damaging events [149]. Depending of the type of lesion, it may be correctly bypassed by RNAP; otherwise, transcriptional stalling or mutagenesis occurs [150]. In fact, stalled RNAP acts as a sensor of DNA damage, where it initiates a transcription-coupled repair that plays a critical role in the maintenance of genome integrity [149]. In addition, RNAPs are active components of cellular DNA damage checkpoints and the initiation of apoptosis [150]. Given their vital role in cellular protein production, RNAPs are an emerging and promising target for chemotherapeutic transcription inhibition in cancer cells [151].

Cornelison et al. demonstrated that the treatment of various ovarian cancer cell lines with the RNAP I inhibitor CX-5461 induced a rapid accumulation of cytosolic DNA and activation of the DDR through ATM/ATR kinases [113]. Transcriptional upregulation of STING was also observed, as well as a cGAS-STING-dependent IRF3 phosphorylation and type I IFN response, both in vitro and in vivo in mouse xenograft ovarian cancer models. DNA damage induced by CX-5461 was previously reported to be a result of its ability to stabilize G-quadruplex DNA structures [152] that block the progression of RNAP I, eventually leading to single-strand DNA gaps or breaks [153].

### 2.3. DNA-Damaging Agents

#### 2.3.1. Ionizing Radiation

The cytotoxicity of ionizing radiation is directly linked to its DNA-damaging activity [154]. The most significant type of DNA damage induced by the fractionated radiation doses conventionally used in radiotherapy is DSB [155], the yield of which increases proportionally with doses starting from as low as a few mGy [156]. While radiation at high doses is known to be highly immunosuppressive owing to the radiosensitivity of the lymphoid system [157], there continues to be emerging evidence that local tumor irradiation can improve both the immunogenicity of tumor cells and promote antitumor immune responses [114]. It logically follows that the DNA fragments, which result from radiation-induced DSB, have been linked with the induction of the cGAS-STING signaling axis (reviewed in [114]). While the resulting type I IFN production within tumor cells or DCs has been shown to facilitate the stimulation of CD8+ T cells for tumor eradiation, there are also undesirable effects that have been associated with radiation-induced cGAS-STING signaling, including increased metastasis and damage to non-cancerous tissues [158]. Ultimately, further investigation is needed to delineate all of the consequences of radiation-induced cGAS-STING activation in the treatment of cancer, and its potential to be exploited for immunotherapeutic purposes.

#### 2.3.2. Alkylating Agents

Alkylating reagents react with and damage DNA through the covalent attachment of an alkyl group on the electron-rich nitrogen atoms of the purine ring of guanine bases [159]. The most common alkyl group added is a single-carbon methyl group; however, longer hydrocarbons can also be added [160]. Monofunctional alkylating agents react with only one strand of the DNA double helix, whereas those that are bifunctional react at sites on both strands to produce a crosslink [161]. When not repaired, either type of lesion will prevent DNA replication during cellular division, and will eventually lead to cell death [162]. In addition to being carcinogenic themselves, most alkylating agents suffer from dose-limiting toxicity to the bone marrow, which is associated with both humoral and cellular immunosuppression [159].

As alluded to earlier, the cGAS-STING pathway is also activated by cytosolic mitochondrial DNA in addition to DNA derived from pathogens or damaging events [163,164]. Maekawa et al. showed that cisplatin treatment of renal tubular cells induced mitochondrial DNA leakage into the cytosol, which subsequently activated cGAS-STING signaling [115]. The resulting STING-dependent inflammatory response was further shown to promote the progression of acute kidney injury in a genetically engineered mouse of model exploiting the known dose-dependent nephrotoxicity of cisplatin [165,166]. Parkes et al. showed that the DNA damage induced by the treatment of HeLa cells with cisplatin at the IC_50_ concentration increased the amount of cytosolic DNA and activated cGAS, as measured by the cytoplasmic levels of the enzyme bound to the Histone H3 immunoprecipitate [116]. The cisplatin treatment increased the mRNA levels of the CXCL10 and CCL5 chemokines in a STING- and cGAS-dependent fashion. Both CXCL10 and CCL5 are known to have an IRF3 binding motif within their promoter regions [162], and their overexpression has been associated with higher levels of CD8+ T cells in melanoma, gastric, and colorectal tumors [167,168,169,170]. The authors also noted increased expression of PD-L1 mRNA. With low doses, Zhou et al. showed that carboplatin induced DNA damage in the H3122 lung cancer cell line, which activated both canonical STING-IRF3 and non-canonical STING-NFκB signaling [117]. This effect was shown to be associated with increased tumor infiltration of CD8+ T cells in a mouse model of Lewis lung carcinoma, which was linked with increased levels of CXCL10 and CCL5 mRNAs. There was also a STING-dependent increase in PD-L1 expression, which significantly improved antitumor immune responses when the alkylating agent was combined with an anti-PD1 treatment.

#### 2.3.3. Nucleoside Analogs

Nucleoside analogs are cytotoxic antimetabolites used as chemotherapeutics and antivirals [171,172]. The cytotoxic activity of these compounds come from their ability to disrupt cellular nucleic acid synthesis via several different mechanisms [171]. In terms of DNA damage, the incorporation of certain nucleoside analogs during replication or repair ultimately leads to stalled replication forks and chain termination [173]. Many nucleoside analogs have significant lymphoid cytotoxicity, resulting in prolonged lymphocyte depletion [174]. While this activity is being exploited for the treatment of some autoimmune disorders, it is a considerable limitation in the context of chemotherapy.

Tian et al. showed that non-cytotoxic doses of the nucleoside analog 5-fluorouracil (5-FU) induced micronuclei-like DNA structures that triggered a cGAS- and STING-dependent type I IFN response in cancer cells [118]. This cancer cell intrinsic response promoted a strong antitumor response in mouse models of colon cancer and melanoma. The authors further showed that antitumor activity was mainly a product of IFN sensing by bone-marrow-derived cells. In addition, it was promoted by a favorable antitumor microenvironment with increased intratumoral T cell levels and reduced numbers of tumor-associated myeloid cells. Wan et al. demonstrated that a micellar nanoparticle system containing the nucleoside analog gemcitabine, conjugated to a PVD polymer, activated cGAS-STING signaling in DCs that increased both antitumor NK and T cell responses in various mouse models of pancreatic cancer [119]. They further showed that activation of STING signaling in tumor cells promoted an induction of the chemokines CCL2 and CCL7 that were associated with immune resistance and was able to be reversed via the incorporation of a CCR2 (CCL2 and CCL7 receptor) antagonist into the formulation.

We recently reported that the pyrimidine nucleoside analog cytarabine (Ara-C) activates the cGAS-STING pathway [120]. We first showed that entrapment of non-cytotoxic doses of Ara-C within a multi-targeted, mannosylated, cationic, liposomal delivery system (DS) greatly improved its biological stability, and that the Ara-C/DS combination had beneficial immunomodulatory properties [175]. This delivery system was designed to specifically target both cancer and immune cells. This prompted us to investigate the immunomodulatory mechanism of Ara-C/DS. We subsequently demonstrated that the DS improves the ability of Ara-C to induce DNA DSB in several human ovarian and colorectal cancer cell lines in vitro, and that this DNA damage leads to the activation of the cGAS-STING signaling axis in an Ara-C dose-dependent fashion [120]. Interestingly, a similar activity was also observed in immune cells including peripheral blood mononuclear cells (PBMCs) and THP-1-derived macrophages. We further demonstrated that Ara-C/DS-mediated DNA damage translated into increased surface expression of various immune ligands on cancer cells, including MHC-1, as well as the NK cell-attracting MHC class 1 chain-related protein A and B (MICA) and UL16-binding proteins 1-62, 5 and 6 (ULBP1-62,5,6) [120], which are important ligands of the NKG2D receptor [176]. These immunomodulatory effects were linked with increased priming of cytotoxic lymphocytes ex vivo using PBMC from colorectal cancer patients, and increased NK cell activity in an in vitro co-culture model. Importantly, the doses of Ara-C used in these studies were shown to be non-toxic to the immune cells [175], and cytostatically reduced cancer cell growth in vitro without directly causing cell death [120].

## 3. Future Perspectives

There is significant immunotherapeutic potential in STING-associated signaling and its ability to promote antitumor immune responses. Overall, this a cumulative effect of the resulting type I IFN and chemokine responses promoting DC maturation [177], the recruitment and activation of both CTL [30,38] and NK cells [178] with requisite increases in cancer cell immune ligand expression (MHC-1, NKG2D) [67,68], and M2 to M1 macrophage repolarization within the TME [59,62,63]. Despite considerable efforts, there remains no clinically approved STING agonist for use in cancer immunotherapy applications, which is a consequence of considerable pharmacokinetic and pharmacodynamic limitations [70,71]. Ultimately, the most critical hurdle for any successful STING agonist immunotherapeutic in the cancer setting is achieving the safe, efficient, and specific delivery in order to induce the necessary localized and acute activation [73,74].

Several recent reports have emerged which suggest that there are potential barriers inherent to the STING pathway that may help to both explain the lack of a successful agonist and guide the development of future strategies. Hong et al. suggested that cGAS-STING-associated inflammatory signaling may actually promote cancer cell survival [179]. They showed that STING-dependent non-canonical NFκB activation in response to chromosomal instability resulted in the activation of the protumor STAT3 transcription factor. STAT3 activation was a product of IL-6 and its receptor (IL-6R), with the use of an IL-6R inhibitor shown to counteract this effect. It remains to be determined whether this effect occurs with agonists that directly bind and activate STING. Li et al. investigated the underlying mechanisms limiting the clinical efficacy of STING agonist therapies, identifying that they promote the proliferation of a particular subset of protumor IL-35+ regulatory B cells in the TME that impaired NK-driven antitumor responses in several preclinical mouse cancer models [180]. The authors linked this effect with an IRF3-dependent and type-I-IFN-independent increase in the expression of the Epstein–Barr-virus-induced gene 3 (Ebi3) that encodes for a subunit of the immunosuppressive IL-35. Combining STING agonism with anti-IL-35 therapy increased NK cell proliferation, reduced tumor volume, and increased animal survival. Zhang et al. linked the canonical activation of NF-κB with prolonged and increased levels of STING signaling, an effect that was a product of inhibited microtubule-mediated Golgi-to-lysosome trafficking [181]. The authors suggested that the cross-talk between the two immunomodulatory pathways may have a cooperative effect in both antibacterial/antiviral and antitumor immune responses. However, the fact that STING pathway activation is itself associated with canonical NF-κB signaling in certain settings warrants consideration, as a possible scenario exists where the use of a STING agonist may promote prolonged activation and detrimental inflammatory effects.

Traditional cancer treatments such as radiation and chemotherapy have well known immunostimulatory effects [84] that may seem counterintuitive at first, given their intended purpose is the eradication of diseased cells. The discovery of the cGAS-STING signaling axis as a critical sensor of cytosolic DNA and initiator of innate immune responses [16] is helping to explain this enigma. When the mechanisms by which these conventional treatments exert their cytotoxic activity are examined, one commonality that emerges is their ability to induce DNA damage. This review has summarized the known reports in which the DNA-damaging activity of traditional cancer therapies has been linked with activation of the cGAS-STING axis. These therapeutic agents can be broadly grouped into three categories, including those that directly damage DNA, inhibitors of proteins involved in the DDR, and those that induce apoptosis-associated DNA damage. It is conceivable that many more of these traditional therapies that act through DNA damage will continue to be linked with the activation of the cGAS-STING pathway.

**Figure 3:**
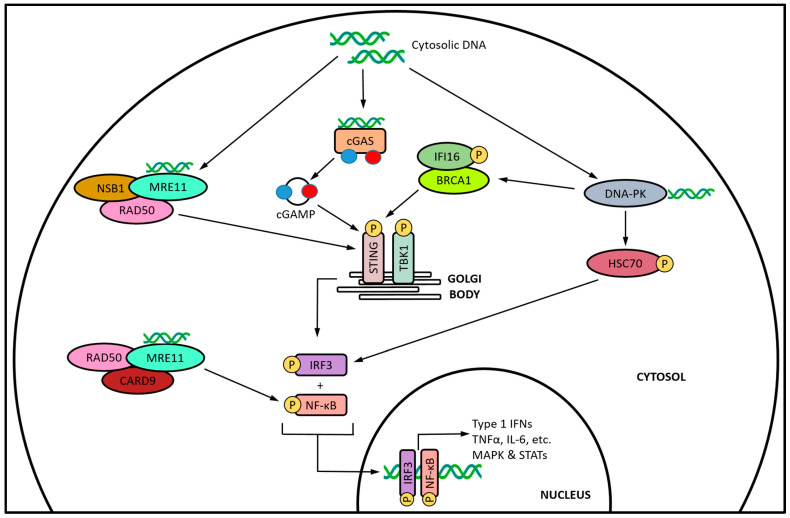
Alternative innate immune signaling pathways activated by cytosolic DNA. In addition to cGAS, the DDR factors DNA-PK and MRE11 facilitate cytosolic DNA sensing, resulting in the activation of both STING itself, and its associated downstream signaling. For simplicity, the activation of endoplasmic-reticulum-bound STING and its translocation to the Golgi have been omitted.

The current lack of approved STING agonist therapies is fueling investigations aimed at developing novel strategies capable of a controlled activation of the pathway. To this end, there is the notion of immunotherapeutic potential in the application of DNA-damaging agents for the indirect activation of STING via stimulating the endogenous production of its prototypical cGAMP ligand. While most of the examples we summarize in this review use cytotoxic doses of the DNA-damaging agent, and note concomitant immunostimulation from STING activation as a secondary by-product, there are a few reports where activation of the pathway is specifically targeted as part of a “chemoimmunotherapy” strategy [99,100]. We believe that there are inherent advantages to the indirect activation of STING through DNA damage. First and foremost, this strategy involves the use of therapeutic agents that already have been clinically approved for their cytotoxic chemotherapy properties, and repurposing them for their immunostimulatory properties at lower than conventional doses. Secondly, stimulating endogenous cGAMP production in the cytosol circumvents the low membrane permeability and intracellular accumulation known to limit the efficacy of direct STING agonists. In addition, there are several cellular mechanisms that function to limit the accumulation of cytosolic DNA, and would act as “built-in” modulation in order to prevent chronic proinflammatory signaling. The aforementioned mechanisms involve the activity of DNA repair proteins, including replication protein A (RPA) and RAD51 that work to prevent the accumulation of cytosolic DNA by binding and retaining DNA fragments within the nuclear compartment [182], as well as the transcription–export (TREX) complex, a 3′-5′ exonuclease that degrades cytosolic DNA in mammalian cells [183]. Finally, targeting STING activation via DNA damage may be an avenue to overcome the known loss of function mutations in STING that will intrinsically limit the efficacy of a direct agonism strategy [184]. Studies have shown that these mutations are highly prevalent in certain populations [185,186,187], where the lack of fully functional STING significantly diminishes signaling downstream of the protein. Cytosolic DNA has the potential to activate associated downstream signaling independent of cGAS and the STING protein. This activation involves several members of the DDR, including DNA-dependent protein kinase (DNA-PK) and the meiotic recombination 11 homolog A (MRE11) nuclease subunit (Figure 3). DNA-PK is a trimeric complex consisting of a catalytic subunit and the Ku70/80 heterodimer, which recognizes the ends of DSB [188]. In addition to being a critical component of the DSB repair system, DNA-PK is also a key DNA sensor that modulates several innate immune pathways [189]. When activated, DNA-PK promotes a STING-dependent innate response via IFN-γ-inducible factor 16 (IFI16) and BRCA1 [190], but can also directly phosphorylate IRF3 independent of STING [191]. MRE11 recognizes and processes DSB as part of HR repair [192], and is known to activate STING-dependent type I IFN production in various cell types [193] as part of the MRN complex along with Nijmegen breakage syndrome protein 1 (NBS1) and the RAD50 ATPase [194]. Together with MRE11, RAD50 also interacts with the caspase-recruitment domain 9 (CARD9) proinflammatory signaling adaptor to directly activate NF-κB, independent of STING [195].

One important concern of any DNA-damage-based immunotherapeutic strategy is the potential for immunotoxicity. While cytotoxicity in cancer cells is acceptable, and even beneficial for the release of antigenic material, this activity must be avoided, or kept to an absolute minimum in hematopoietic cells, in order to avoid the adverse immunological effects that plague these agents when used at conventional doses. Continued advancements in the development and use of targeted nanoparticle delivery systems will undoubtedly aid in this endeavor. Indeed, the two related reports of Cao et al. and Zhao et al. utilize nanoparticle delivery systems to target cytotoxic doses of the respective chemotherapeutics to cancer cells, with subsequent paracrine activation of STING in tumor resident immune cells [99,100]. Our own work with the Ara-C/DS follows a similar strategy, albeit with a few important distinctions. We have shown that the multi-targeted nature of the DS leads to DNA damage and cGAS-STING axis activation in both cancer cells and immune cells, with Ara-C doses that are non-cytotoxic to the latter [120,175]. Ultimately, the potential implications and/or benefits of this dual activity requires further investigation.

## 4. Conclusions

The role of DNA damage in the activation of immune responses continues to be delineated, from which the cGAS-STING pathway has been identified as serving a critical role. The highly touted potential of this signaling axis for cancer immunotherapy remains to be exploited. As complexities inherent to the pathway continue to be discovered, they will undoubtedly help to both rationalize the lack of an approved STING agonist therapy, and guide measures to circumvent these obstacles. The link between the DNA-damaging activities of traditional cancer therapies and cGAS-STING activation that we have reviewed herein is driving investigations into their application in the cancer immunotherapy setting. While there are several potential advantages inherent to such an indirect approach, one of the major concerns and potentially limiting factors is the associated activation of inflammatory pathways that can hinder immunotherapeutic efficacy, and even promote cancer growth [196,197,198,199]. The applicability of using DNA-damaging agents for immunotherapeutic purposes will certainly need to be demonstrated. We believe that this may be progressed through the use of low doses of DNA-damaging agents, coupled with advancements in targeted nanoparticle delivery systems. Together, they aim to minimize inflammatory responses while maintaining the beneficial immunostimulatory effects. Ultimately, what is clear is that any successful STING agonism strategy will need to balance the duality of the contrasting antitumor with the protumor/inflammatory properties inherent to the targeted pathway.

## Figures and Tables

**Table 1:** Summary of conventional cancer therapies linked with cGAS-STING signaling axis activation.

Category	Class	Mechanism of DNA Damage	Agent	Dose	Reference
Antimitotic Agents	Microtubule Targeting Agents	Induction of apoptosis-associated nucleases; Disruption of intracellular DNA repair protein trafficking.	Taxol (Paclitaxel) Eribulin	Cytotoxic Cytotoxic	[94] [95] [96] [97]
DDR Enzyme Inhibitors	Topoisomerase Inhibitors	Disruption of enzyme catalyzed changes in DNA double helix structure during replication, transcription and repair.	Camptothecin	Non-Cytotoxic	[98]
			Camptothecin + Cisplatin	Cytotoxic	[99]
			SN38	Cytotoxic	[100]
			Topotecan	Cytotoxic	[101]
			Doxorubicin, Daunorubicin	Non-Cytotoxic	[102]
			Etoposide	Non-Cytotoxic Cytotoxic	[103] [104]
	PARP Inhibitors	Disruption of enzyme catalyzed synthesis of Poly(ADP-ribose) signal for other DNA-repairing enzymes.	Olaparib	Cytotoxic	[105]
					[106]
					[107]
				N/A	[108]
			Rucaparib	Cytotoxic	[105]
			Talazoparib	Non-Cytotoxic (Cytostatic)	[109]
	ATR and ATM Inhibitors	Indirect DNA damage from inhibition of DDR; Disruption of coordination between cell cycle and DDR.	Ceralasertib (AZD6738) KU60019 AZD1390 and KU55933	N/A N/A N/A	[110] [111] [112]
	RNA Polymerase Inhibitors	Nucleolar disruption from blocking of transcription initiation and promoter release.	CX-5461	N/A	[113]
Direct DNA-Damaging Agents	Radiotherapy	Base pair damage, single- and double-strand breaks from high energy of radiation.	Ionizing Radiation	N/A	Reviewed in [114]
	Alkylating Agents	Covalent attachment of alkyl groups to guanine base purine rings which interferes with enzymes involved in replication and transcription.	Cisplatin	Cytotoxic	[115] [116]
			Cisplatin + Camptothecin	Cytotoxic	[99]
			Carboplatin	Non-Cytotoxic	[117]
	Nucleoside Analogs	Disruption of cellular nucleic acid synthesis; Incorporation into DNA sequence leading to stalled replication forks.	5-Fluorouracil	Non-Cytotoxic	[118]
			Gemcitabine	N/A	[119]
			Cytarabine	Non-Cytotoxic (Cytostatic)	[120]

Dose designation based on whether or not direct evidence was presented of cytotoxicity (in vitro cell death and/or in vivo tumor reduction/cell death) with the particular therapy at the specific doses(s) linked with cGAS-STING activation. Cases where this designation could not be made have been assigned N/A for not available. PARP: poly(ADP-ribose) polymerase; ATR: ataxia telangiectasia and Rad3-related; ATM: ataxia telangiectasia mutated.

## Data Availability

No new data were created or analyzed in this study. Data sharing is not applicable to this article.

## Supplementary Materials

The following supporting information can be downloaded at: https://www.mdpi.com/article/10.3390/cancers15164127/s1, Table S1: Chemical structures of conventional cancer therapeutics linked to cGAS-STING activation.
