# Supplementary material for: Conventional DNA-Damaging Cancer Therapies and Emerging cGAS-STING Activation: A Review and Perspectives Regarding Immunotherapeutic Potential
Source: Cancers (Basel). 2023 Aug 16;15(16):4127. doi: 10.3390/cancers15164127 (PMC10453198; doi:10.3390/cancers15164127)
Supplement: Supplementary file 1 [file cancers-15-04127-s001.zip › cancers-2527644-supplementary.pdf]

SUPPLEMENTAL INFORMATION

Conventional DNA Damaging Cancer Therapies and Emerging cGAS-STING Activation: A Review and Perspectives of Immunotherapeutic Potential

Jordan D. Lewicky [1], Alexandrine L. Martel [1], Mukul Raj Gupta [2], René Roy [2], Galaxia M. Rodriguez [3,4], Barbara C. Vanderhyden [3,4], Hoang-Thanh Le [1,5,6*]

[1] Health Sciences North Research Institute, 56 Walford Road, Sudbury, ON P3E 2H2, Canada; jlewicky@hsnri.ca (J.D.L.); amartel@hsnri.ca (A.L.M.)
[2] Glycosciences and Nanomaterial Laboratory, Université du Québec à Montréal, P.O. Box 8888, Succ. Centre-Ville, Montréal, QC H3C 3P8, Canada; guptamukulraj@gmail.com (M.K.); roy.rene@uqam.ca (R.R)
[3] Cancer Therapeutics Program, Ottawa Hospital Research Institute, 501 Smyth Rd., Ottawa, ON K1H 8L6, Canada; garodriguez@ohri.ca (G.M.R.); bvanderhyden@ohri.ca (B.C.V.)
[4] Department of Cellular and Molecular Medicine, University of Ottawa, 451 Smyth Rd., Ottawa, ON K1H 8M5, Canada.
[5] Medicinal Sciences Division, NOSM University, 935 Ramsey Lake Road, Sudbury, ON P3E 2C6, Canada.
[6] School of Natural Sciences, Laurentian University, 935 Ramsey Lake Road, Sudbury, ON P3E 2C6, Canada.
* Correspondence: hle@hsnri.ca (H.-T.L)

Table S1 2

Table S1. Chemical structures of conventional cancer therapeutics linked with cGAS-STING activation.

Category	Class	Structures
Antimitotic Agents	Microtubule Targeting Agents	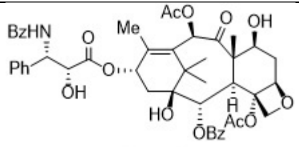 Taxol
DDR Enzyme Inhibitors	Topoisomerase Inhibitors	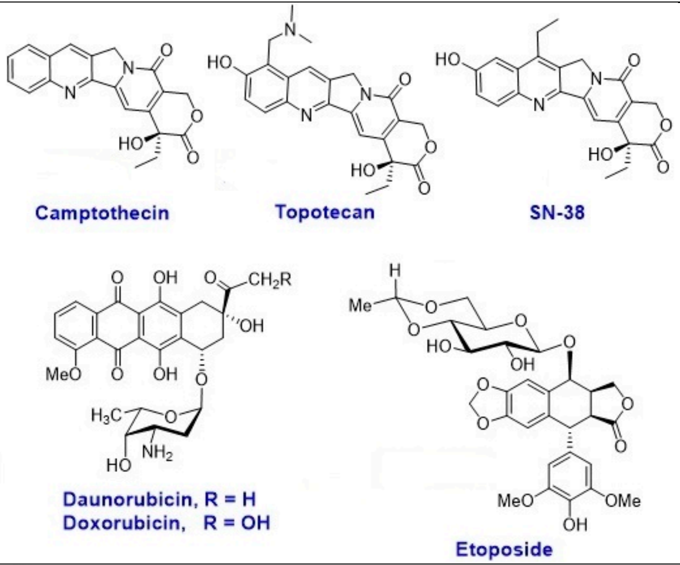
	PARP Inhibitors	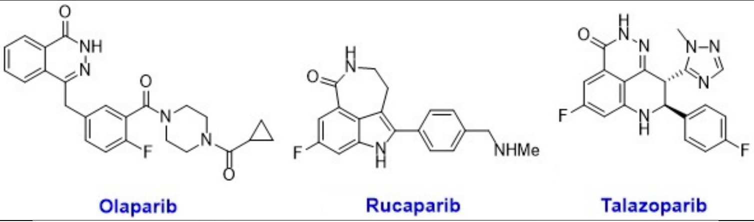
	ATR & ATM Inhibitors	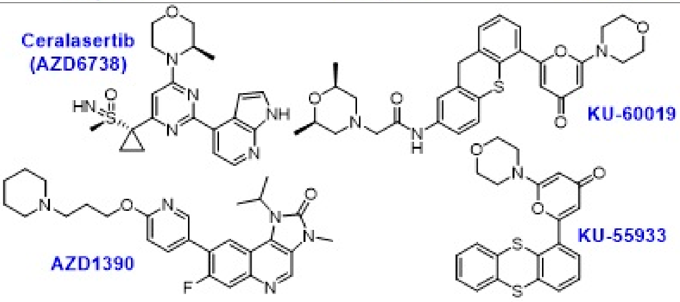
Direct DNA Damaging Agents	Alkylating Agents<br><br>Nucleoside Analogs	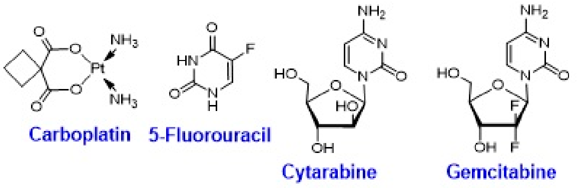
